# Supplementary material for: A method to synthesize analytical rhodoquinone standards for quantitative analysis in tissue specimen
Source: J Lipid Res. 2026 Jul 9;67(8):101099. doi: 10.1016/j.jlr.2026.101099 (PMC13470162; doi:10.1016/j.jlr.2026.101099)
Supplement: Supplementary file 1 [file mmc1.pdf]

Figure S1

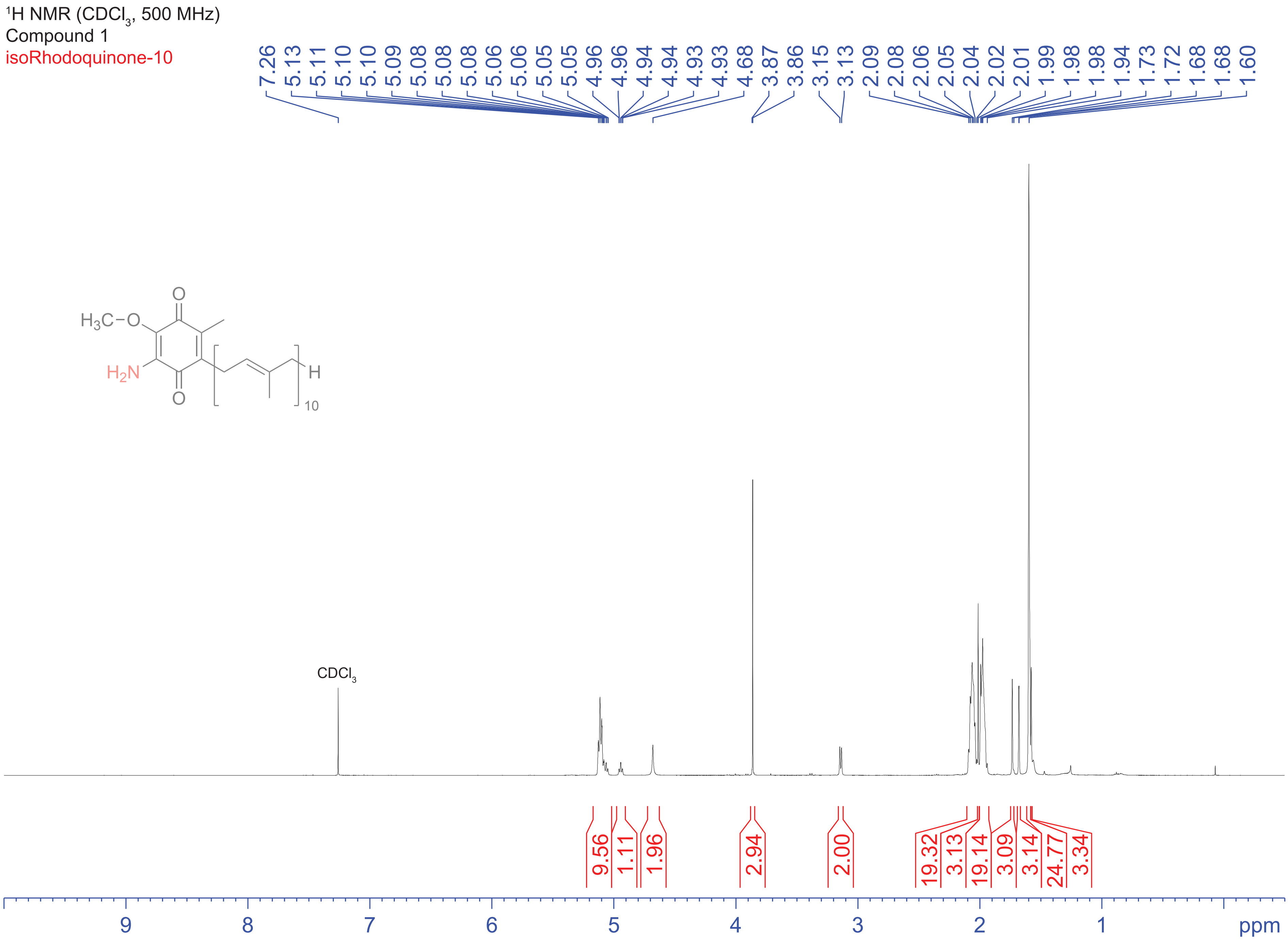

1H NMR (CDCl3, 500 MHz)
Compound 1
isoRhodoquinone-10
7.26 5.13 5.11 5.10 5.10 5.09 5.08 5.08 5.08 5.06 5.06 5.05 5.05 4.96 4.96 4.94 4.94 4.93 4.93 4.68 3.87 3.86 3.15 3.13 2.09 2.08 2.06 2.05 2.04 2.02 2.01 1.99 1.98 1.98 1.94 1.73 1.72 1.68 1.68 1.60
H3C−O
H2N
O
H
10
CDCl3
9.56 1.11 1.96 2.94 2.00 19.32 3.13 19.14 3.09 3.14 24.77 3.34
9 8 7 6 5 4 3 2 1 ppm

Figure S2

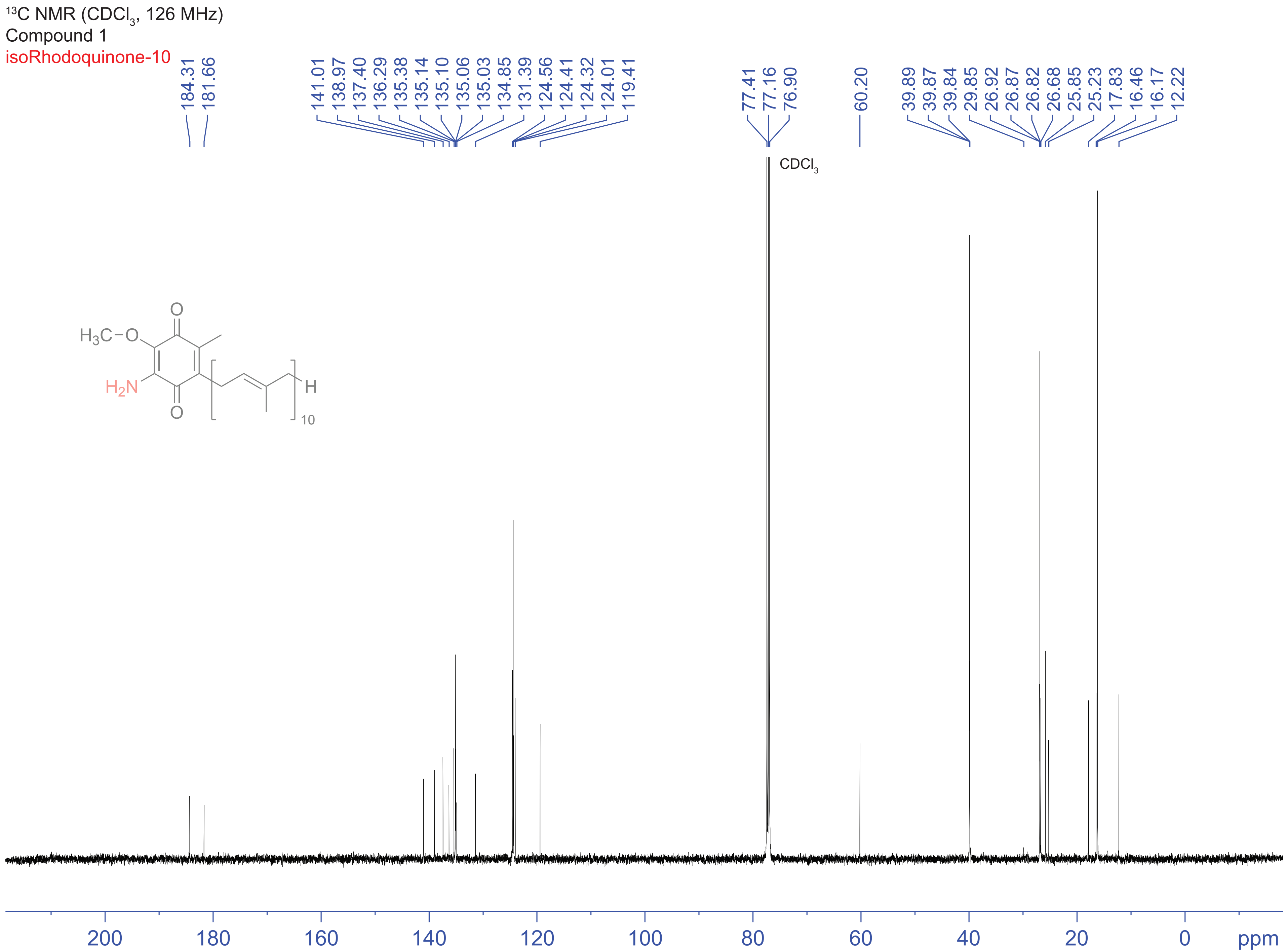
^{13}C NMR (CDCl$_3$, 126 MHz)
Compound 1
isoRhodoquinone-10
184.31
181.66
141.01
138.97
137.40
136.29
135.38
135.14
135.10
135.06
135.03
134.85
131.39
124.56
124.41
124.32
124.01
119.41
77.41
77.16
76.90
60.20
39.89
39.87
39.84
29.85
26.92
26.87
26.82
26.68
25.85
25.23
17.83
16.46
16.17
12.22
CDCl$_3$
H_3C-O
H_2N
O
O
H
10
200
180
160
140
120
100
80
60
40
20
0
ppm

Figure S3

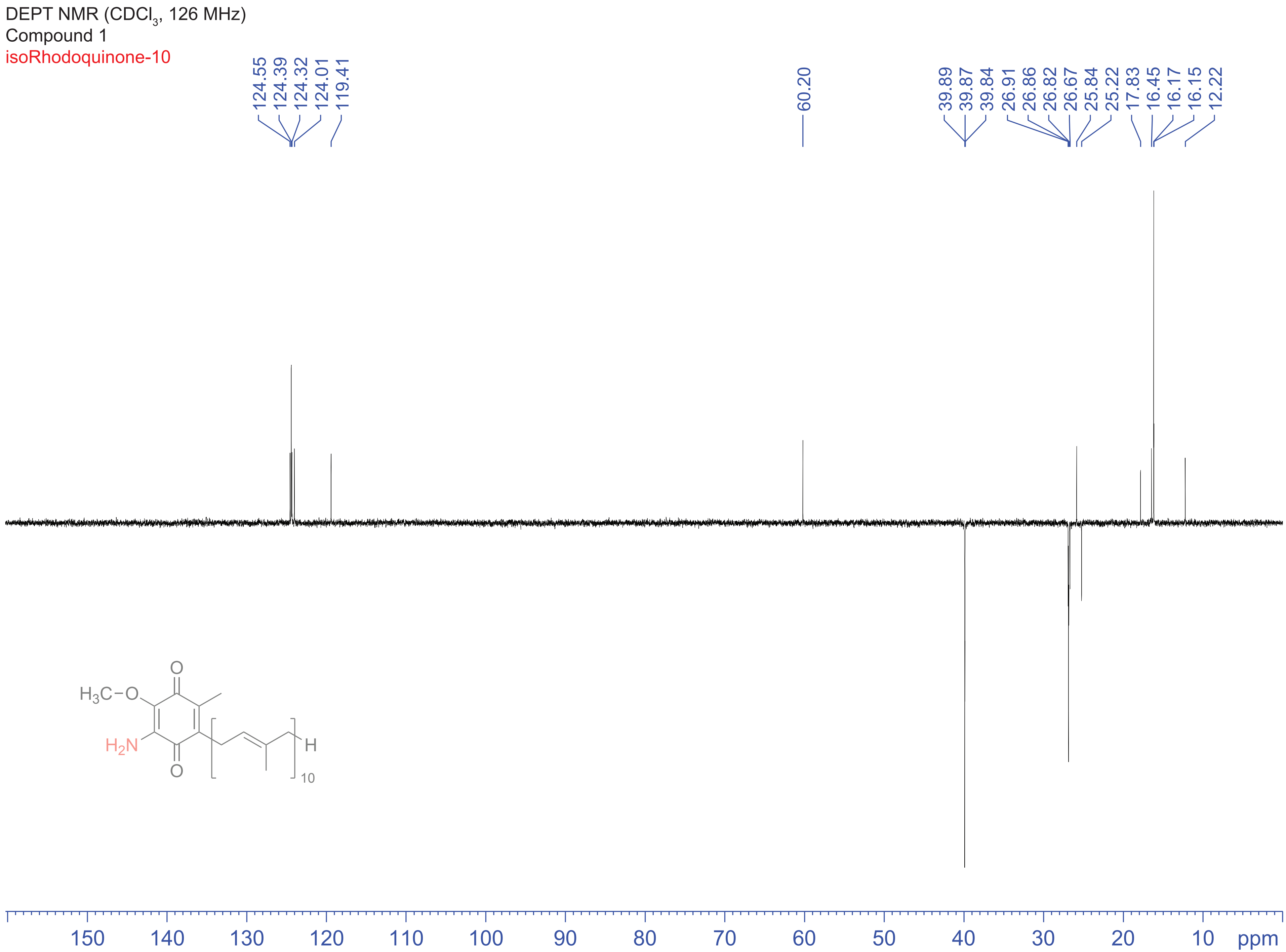
DEPT NMR (CDCl3, 126 MHz)
Compound 1
isoRhodoquinone-10
124.55
124.39
124.32
124.01
119.41
60.20
39.89
39.87
39.84
26.91
26.86
26.82
26.67
25.84
25.22
17.83
16.45
16.17
16.15
12.22
H3C−O
O
O
H2N
H
10
150
140
130
120
110
100
90
80
70
60
50
40
30
20
10
ppm

Figure S4

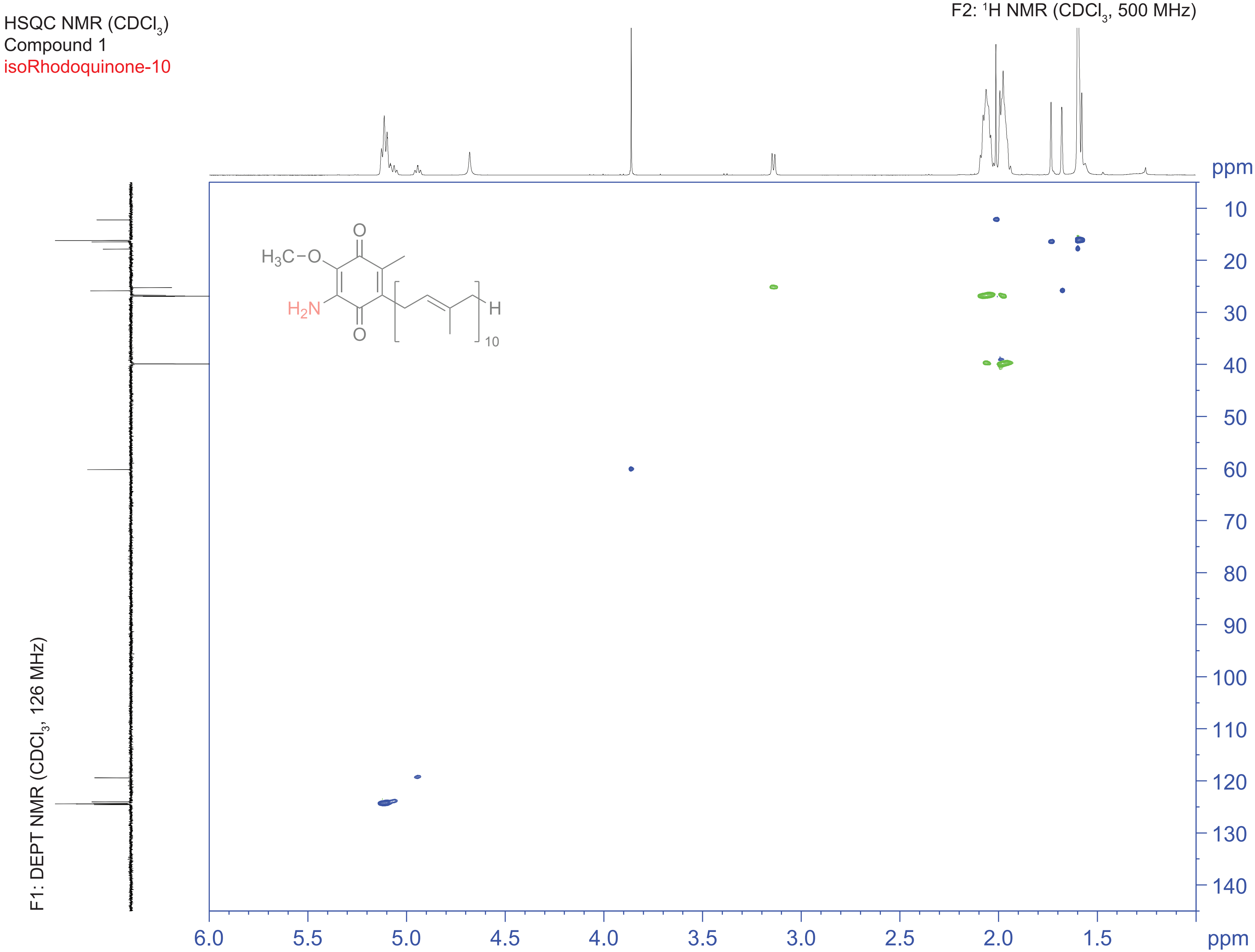

HSQC NMR (CDCl3)
Compound 1
isoRhodoquinone-10
F2: 1H NMR (CDCl3, 500 MHz)
F1: DEPT NMR (CDCl3, 126 MHz)
H3C−O
H2N
O
O
H
10
ppm
10
20
30
40
50
60
70
80
90
100
110
120
130
140
6.0
5.5
5.0
4.5
4.0
3.5
3.0
2.5
2.0
1.5
ppm

Figure S5

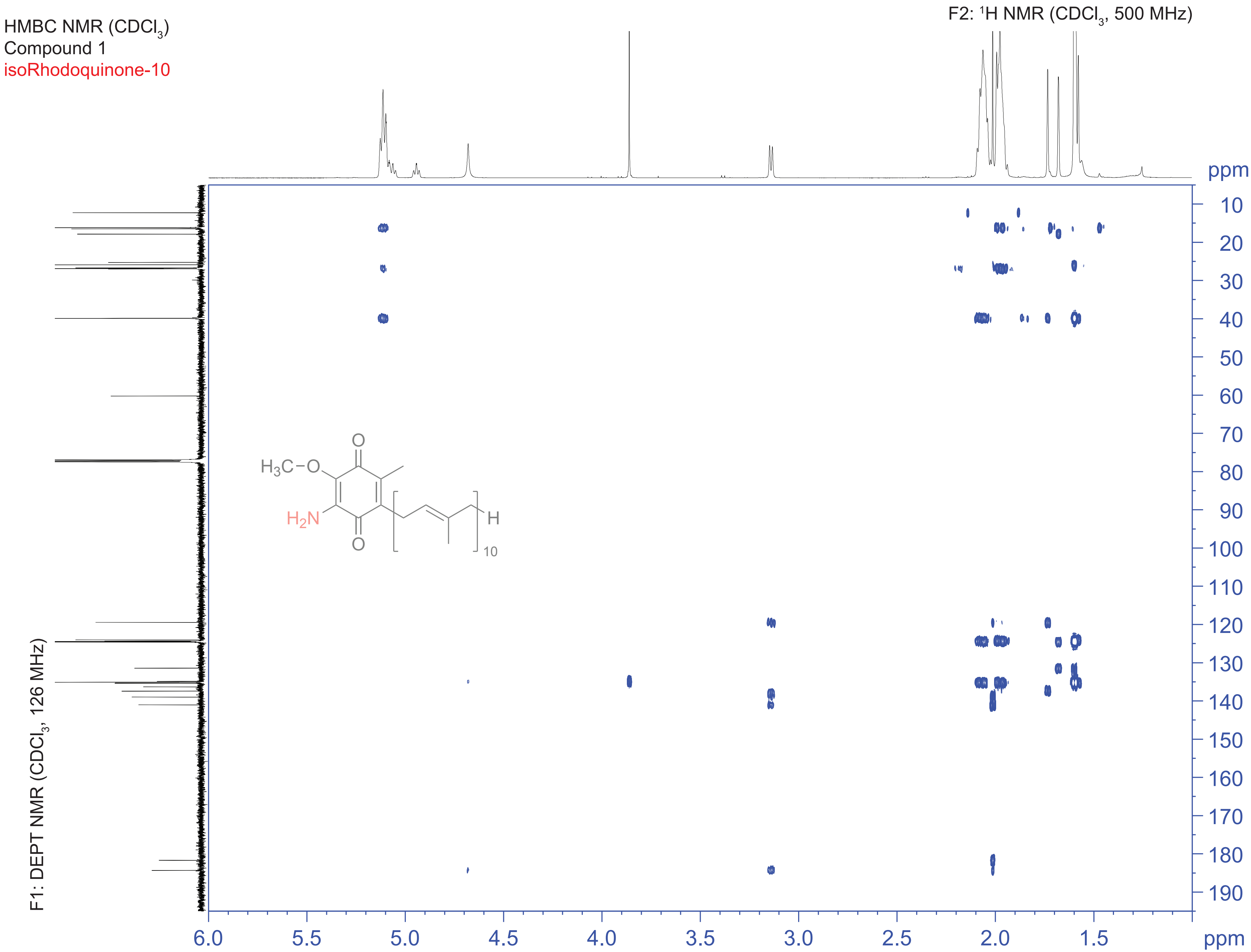
HMBC NMR ($CDCl_3$)
Compound 1
isoRhodoquinone-10
F2: ^1H NMR ($CDCl_3$, 500 MHz)
F1: DEPT NMR ($CDCl_3$, 126 MHz)
ppm
10
20
30
40
50
60
70
80
90
100
110
120
130
140
150
160
170
180
190
6.0
5.5
5.0
4.5
4.0
3.5
3.0
2.5
2.0
1.5
ppm
H_3C-O
O
O
H_2N
H
10

Figure S6

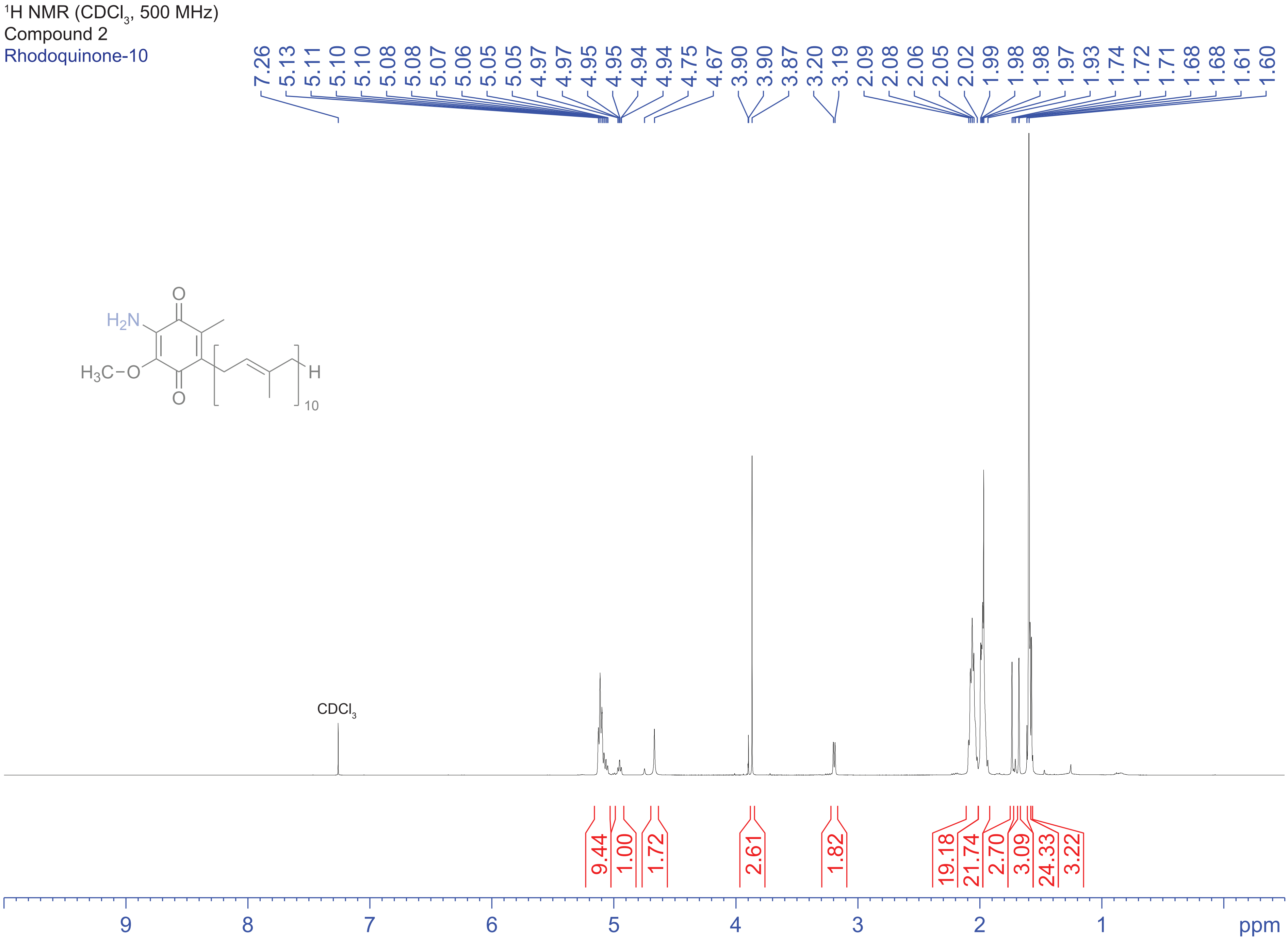

¹H NMR (CDCl3, 500 MHz)
Compound 2
Rhodoquinone-10
7.26 5.13 5.11 5.10 5.10 5.08 5.08 5.07 5.06 5.05 5.05 4.97 4.97 4.95 4.95 4.94 4.94 4.75 4.67 3.90 3.90 3.87 3.20 3.19 2.09 2.08 2.06 2.05 2.02 1.99 1.98 1.98 1.97 1.93 1.74 1.72 1.71 1.68 1.68 1.61 1.60
H2N
H3C–O
O
H
10
CDCl3
9.44 1.00 1.72 2.61 1.82 19.18 21.74 2.70 3.09 24.33 3.22
9 8 7 6 5 4 3 2 1 ppm

Figure S7

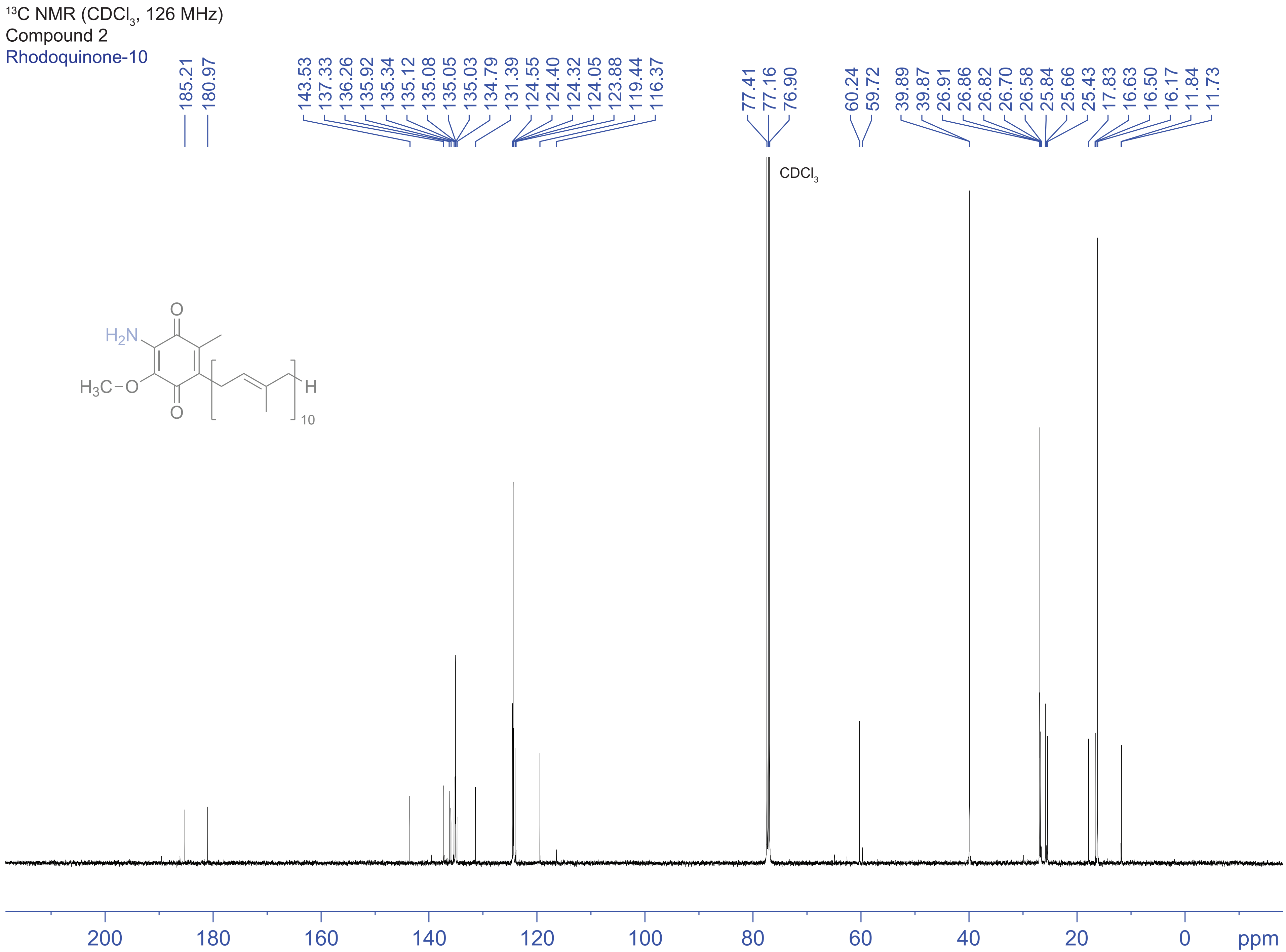

¹³C NMR (CDCl₃, 126 MHz)
Compound 2
Rhodoquinone-10
185.21
180.97
143.53
137.33
136.26
135.92
135.34
135.12
135.08
135.05
135.03
134.79
131.39
124.55
124.40
124.32
124.05
123.88
119.44
116.37
77.41
77.16
76.90
60.24
59.72
39.89
39.87
26.91
26.86
26.82
26.70
26.58
25.84
25.66
25.43
17.83
16.63
16.50
16.17
11.84
11.73
CDCl₃
H₂N
H₃C−O
O
O
H
10
200
180
160
140
120
100
80
60
40
20
0
ppm

Figure S8

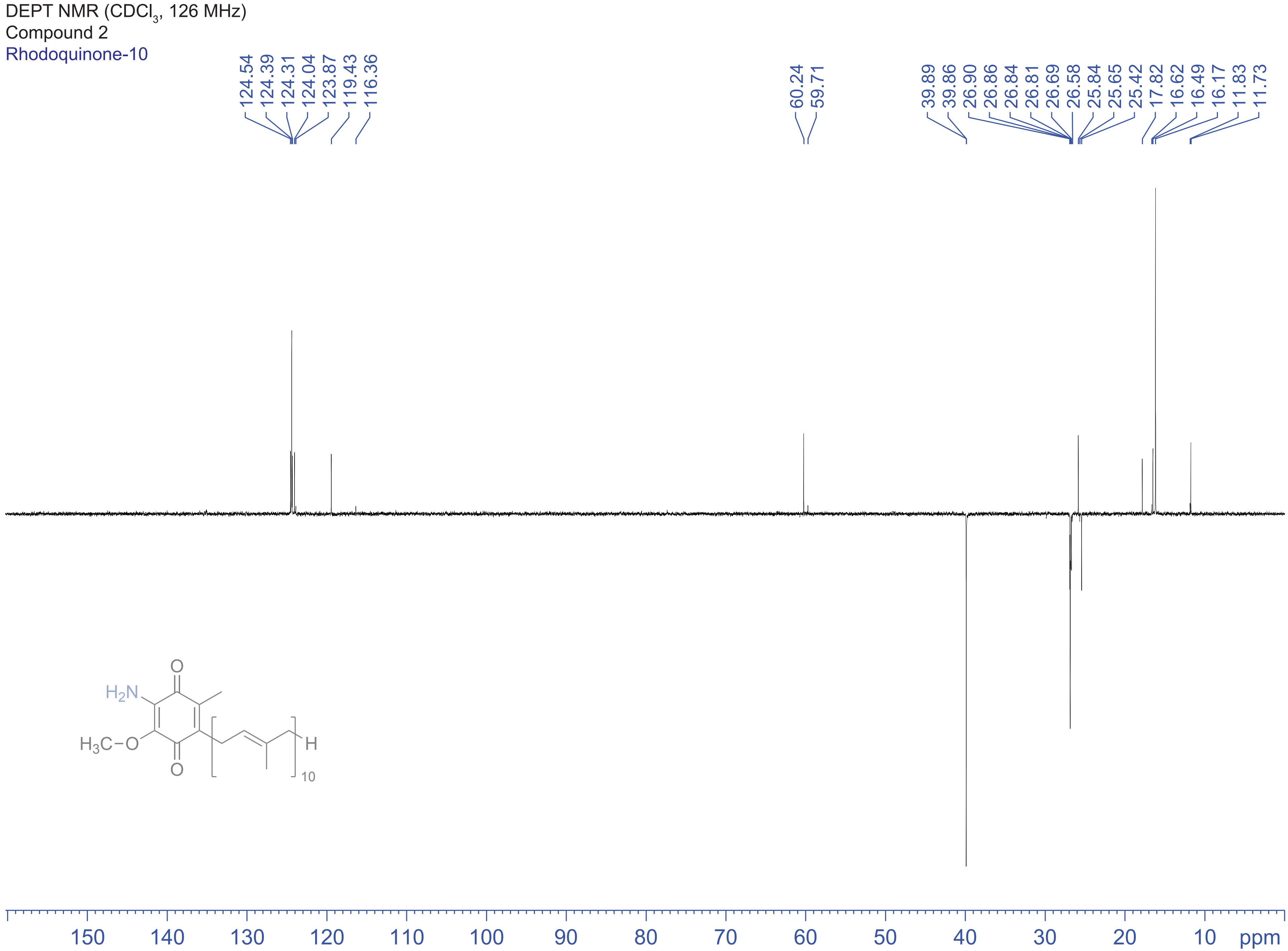

DEPT NMR ($CDCl_3$, 126 MHz)
Compound 2
Rhodoquinone-10
124.54
124.39
124.31
124.04
123.87
119.43
116.36
60.24
59.71
39.89
39.86
26.90
26.86
26.84
26.81
26.69
26.58
25.84
25.65
25.42
17.82
16.62
16.49
16.17
11.83
11.73
O
H_2N
H_3C−O
O
H
10
150
140
130
120
110
100
90
80
70
60
50
40
30
20
10
ppm

Figure S9

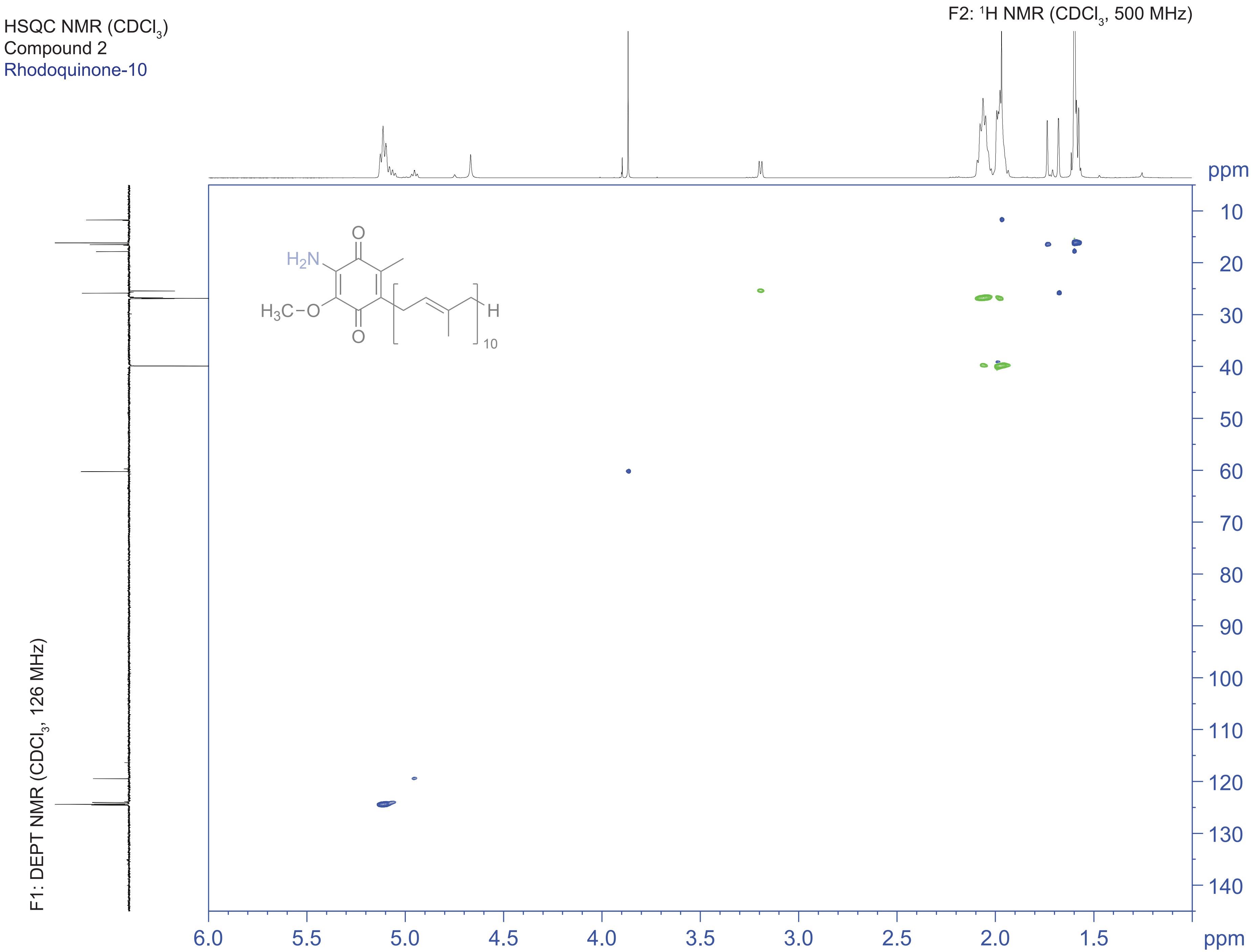
HSQC NMR ($CDCl_3$)
Compound 2
Rhodoquinone-10
F2: ^1H NMR ($CDCl_3$, 500 MHz)
F1: DEPT NMR ($CDCl_3$, 126 MHz)
H_2N
H_3C-O
O
H
10
ppm
10
20
30
40
50
60
70
80
90
100
110
120
130
140
6.0
5.5
5.0
4.5
4.0
3.5
3.0
2.5
2.0
1.5
ppm

Figure S10

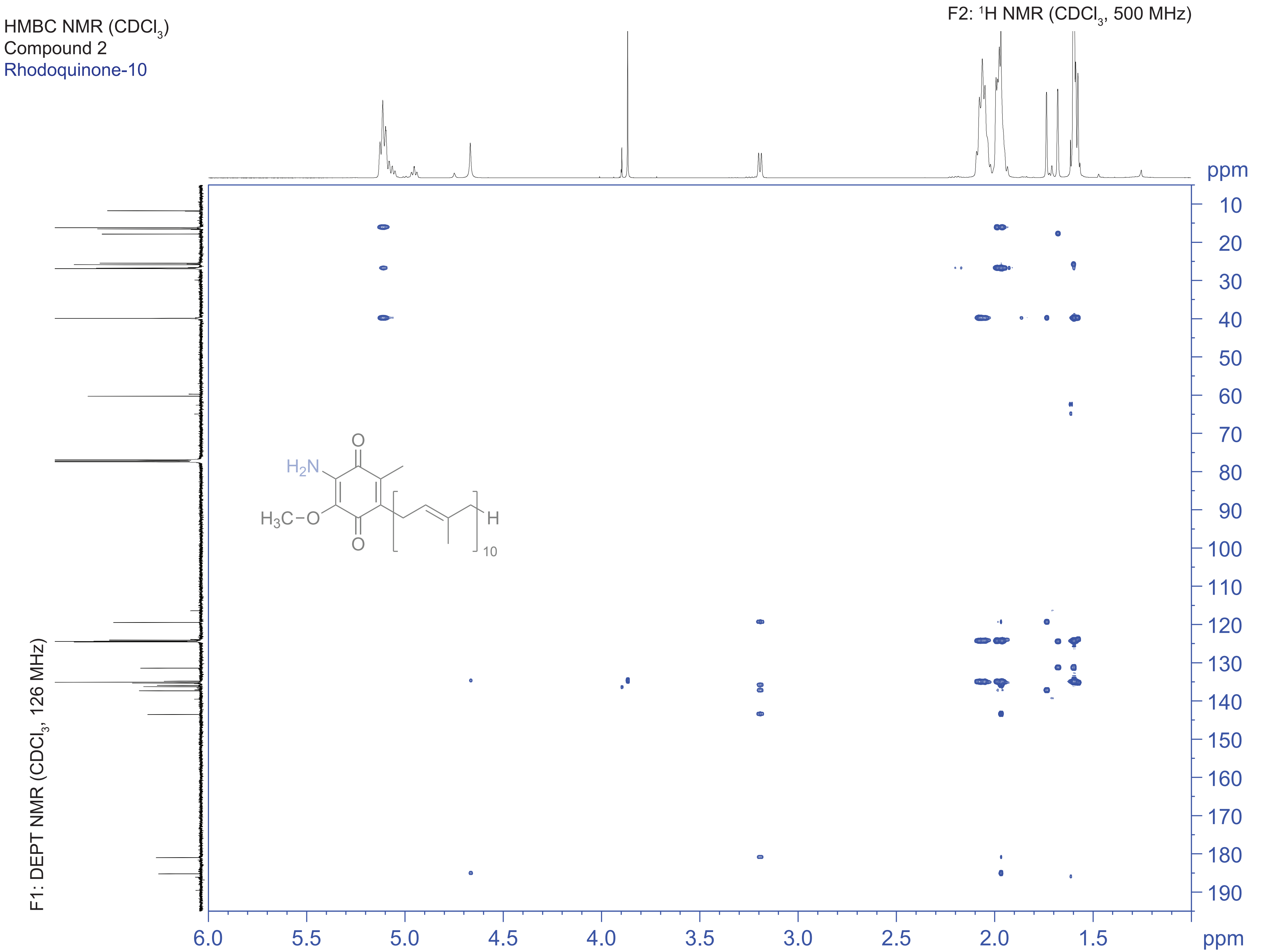

HMBC NMR ($CDCl_3$)
Compound 2
Rhodoquinone-10
F2: ^{1}H NMR ($CDCl_3$, 500 MHz)
F1: DEPT NMR ($CDCl_3$, 126 MHz)
ppm
10
20
30
40
50
60
70
80
90
100
110
120
130
140
150
160
170
180
190
6.0
5.5
5.0
4.5
4.0
3.5
3.0
2.5
2.0
1.5
ppm
O
H_2N
H_3C-O
O
H
10

Figure S11

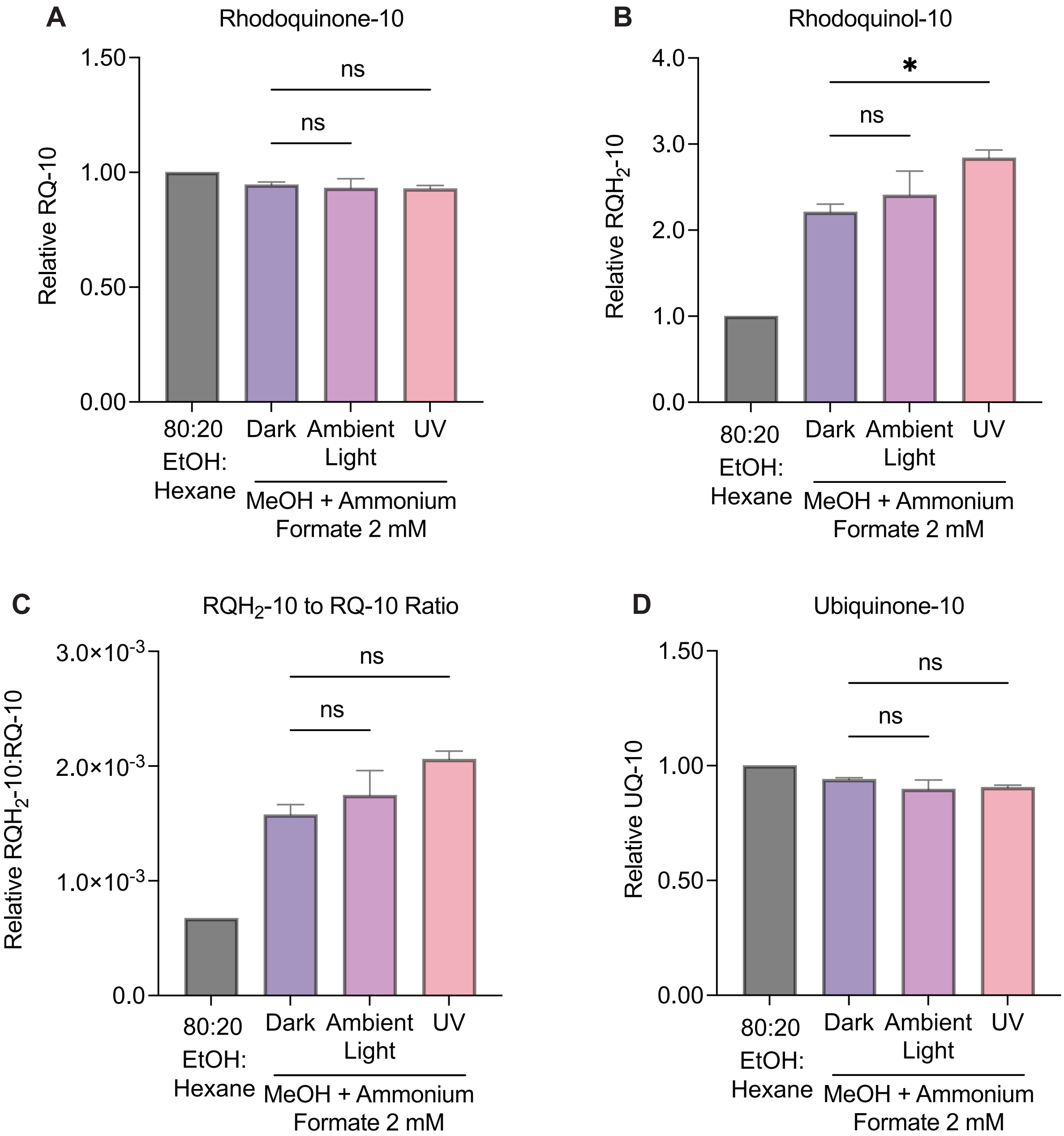
A
Rhodoquinone-10
Relative RQ-10
1.50
1.00
0.50
0.00
ns
ns
80:20 EtOH: Hexane
Dark
Ambient Light
UV
MeOH + Ammonium Formate 2 mM
B
Rhodoquinol-10
Relative RQH2-10
4.0
3.0
2.0
1.0
0.0
*
ns
80:20 EtOH: Hexane
Dark
Ambient Light
UV
MeOH + Ammonium Formate 2 mM
C
RQH2-10 to RQ-10 Ratio
Relative RQH2-10:RQ-10
3.0×10-3
2.0×10-3
1.0×10-3
0.0
ns
ns
80:20 EtOH: Hexane
Dark
Ambient Light
UV
MeOH + Ammonium Formate 2 mM
D
Ubiquinone-10
Relative UQ-10
1.50
1.00
0.50
0.00
ns
ns
80:20 EtOH: Hexane
Dark
Ambient Light
UV
MeOH + Ammonium Formate 2 mM

Figure S12

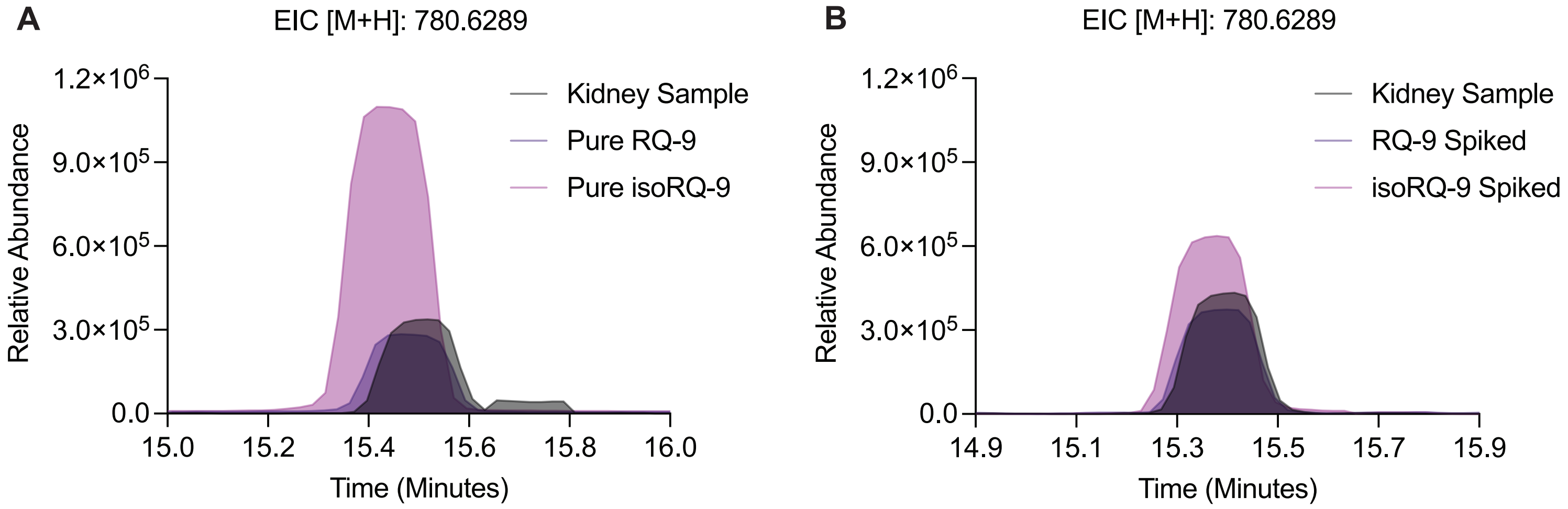
A
EIC [M+H]: 780.6289
1.2×10^6
9.0×10^5
6.0×10^5
3.0×10^5
0.0
Relative Abundance
15.0
15.2
15.4
15.6
15.8
16.0
Time (Minutes)
Kidney Sample
Pure RQ-9
Pure isoRQ-9
B
EIC [M+H]: 780.6289
1.2×10^6
9.0×10^5
6.0×10^5
3.0×10^5
0.0
Relative Abundance
14.9
15.1
15.3
15.5
15.7
15.9
Time (Minutes)
Kidney Sample
RQ-9 Spiked
isoRQ-9 Spiked
